# Organism-Sediment Interactions Govern Post-Hypoxia Recovery of Ecosystem Functioning

**DOI:** 10.1371/journal.pone.0049795

**Published:** 2012-11-21

**Authors:** Carl Van Colen, Francesca Rossi, Francesc Montserrat, Maria G. I. Andersson, Britta Gribsholt, Peter M. J. Herman, Steven Degraer, Magda Vincx, Tom Ysebaert, Jack J. Middelburg

**Affiliations:** 1 Department of Biology, Marine Biology Section, Ghent University, Ghent, Belgium; 2 Netherlands Institute for Sea Research (NIOZ-Yerseke), Yerseke, The Netherlands; 3 Ecologie des systèmes marins côtiers (Ecosysm) UMR 5119 CNRS-Université Montpellier 2-IRD-Ifremer Place Eugène Bataillon, Université Montpellier II, Case 093, F-34095 Montpellier, France; 4 Management Unit of the of the North Sea Mathematical Model, Marine Ecosystem Management Section, Royal Belgian Institute of Natural Sciences, Brussels, Belgium; 5 Wageningen University, Institute for Marine Resources & Ecosystem Studies, Yerseke, The Netherlands; 6 Faculty of Geosciences, Utrecht University, Utrecht, The Netherlands; Institute of Marine Research, Norway

## Abstract

Hypoxia represents one of the major causes of biodiversity and ecosystem functioning loss for coastal waters. Since eutrophication-induced hypoxic events are becoming increasingly frequent and intense, understanding the response of ecosystems to hypoxia is of primary importance to understand and predict the stability of ecosystem functioning. Such ecological stability may greatly depend on the recovery patterns of communities and the return time of the system properties associated to these patterns. Here, we have examined how the reassembly of a benthic community contributed to the recovery of ecosystem functioning following experimentally-induced hypoxia in a tidal flat. We demonstrate that organism-sediment interactions that depend on organism size and relate to mobility traits and sediment reworking capacities are generally more important than recovering species richness to set the return time of the measured sediment processes and properties. Specifically, increasing macrofauna bioturbation potential during community reassembly significantly contributed to the recovery of sediment processes and properties such as denitrification, bedload sediment transport, primary production and deep pore water ammonium concentration. Such bioturbation potential was due to the replacement of the small-sized organisms that recolonised at early stages by large-sized bioturbating organisms, which had a disproportionately stronger influence on sediment. This study suggests that the complete recovery of organism-sediment interactions is a necessary condition for ecosystem functioning recovery, and that such process requires long periods after disturbance due to the slow growth of juveniles into adult stages involved in these interactions. Consequently, repeated episodes of disturbance at intervals smaller than the time needed for the system to fully recover organism-sediment interactions may greatly impair the resilience of ecosystem functioning.

## Introduction

To date most ecosystems are profoundly affected by multiple human activities that alter the systems’ diversity, functioning and delivered services, e.g. [1], [2]. These activities range from single and recurring disturbances to continuous stress of which the consequences for ecosystem resilience (i.e. movement within and between stability domains) may depend on the magnitude of induced change and the diversity-stability relationships that occur during recovery [3]. In order to assess ecosystem resilience, it is useful to measure community dynamics from disturbance-recovery experiments, as recovery reveals the processes determining the response of ecological systems to changes in disturbance regime [4]. Ecological theory indicates that increased levels of biodiversity often result in increased ecosystem functioning [5], [6], which may insure stability against environmental change via compensatory processes and species performance-enhancing effects [7], [8]. Nonetheless, the magnitude and direction of the biodiversity-ecosystem function relationship was also shown to be idiosyncratic and depending on the disturbance context [9]–[12]. Moreover, often one or few particular species have a disproportionate influence on ecosystem properties [13], [14], depending on its functional traits [15]–[18]. Additionally, changes in species interactions and population dynamics during the recovery process are likely to affect the performances of particular ecosystem functions. Consequently, ecosystem resilience may depend on the time needed to restore ecosystem functioning by post-disturbance reassembling of those species with particular functional traits that have distinct effects on ecosystem processes.

Depleted oxygen conditions are becoming increasingly common in coastal areas and represent an important problem for the present coastal benthic environments [19], [20]. Hypoxia occurs naturally in some bottom waters due to limited circulation (e.g. fjords), riverine inputs of organic matter in coastal embayments (e.g. estuaries), or up-welling of subsurface oxygen depleted waters (e.g. shelf systems) [21]. In addition, there is strong evidence for a global increase in the frequency, extent, and intensity of hypoxic events owing to predicted future increases in eutrophication and water-column stratification resulting from sea water temperature rise [19]. For example, nutrient enrichment results in an enhanced occurrence of algal blooms which are known to cause hypoxic conditions in sheltered coastal habitats, such as tidal flats, through decompositional processes and a reduced exchange of oxygen with the water column [22], [23]. Consequences of hypoxia are multiple, including changes in organisms’ behavior and physiology, a loss in biodiversity and altered ecosystem functioning [24]–[27]. However, it is unclear how hypoxic events will affect ecosystem resilience because knowledge on synergistic recovery mechanisms and their time scales is lacking, e.g. how recovering benthic diversity interacts with the stock of reduced compounds that accumulate during oxygen depletion [27]–[29].

Macrofaunal organisms affect sediment functioning in coastal benthic soft-sediment systems through bio-irrigation and bioturbation activities [30]–[33]. These activities mainly depend on size, mobility, and species’ sediment reworking traits which are often associated with feeding activities [18], and are known to alter sediment properties and structure, thereby creating spatial heterogeneity which may facilitate or inhibit other species and hence affect diversity (i.e. ecosystem engineering, [34]). Fore example, these organism-sediment interactions greatly modify sediment biogeochemistry and affect microbial organisms [35]–[36] that are the primary remineralizers of organic matter and the main drivers of biogeochemical processes in coastal sediments [37], [38].

In order to assess benthic community responses to hypoxia, the present study aims at understanding the dynamics of the relationship between reassembling macrobenthos communities and sediment properties determining ecosystem functioning following hypoxia. We therefore evaluate the relation between the time scale of returning community traits (i.e. diversity, abundance, biomass, bioturbation potential *sensu* Solan et al. [18]) and the return rates of several ecosystem processes following experimentally-induced hypoxia in a coastal tidal flat. Macrofaunal recovery typically involves successional dynamics which are strongly determined by the temporal and spatial scale of disturbance [39] with slowest return rates (i.e. years-decades) in the largest disturbed areas with limited ecological connectivity [40]. In contrast, return rates of microbial communities are fast, ranging from hours to a few months, depending on the type of disturbance [41]–[43]. However, if indirect sediment modifications by macrofaunal bioturbation and bio-irrigation activities are important, we expected that the return rate of typically microbial-mediated ecosystem processes would differ from the ‘microbial’ time scale and rather follow the macrofaunal return rate instead.

## Materials and Methods

### Site Description and Experimental Design

The experiment was conducted at an intertidal mudflat (Paulinapolder, 51° 21′ 24′′ N, 3° 42′ 51′′ E) located within the polyhaline part of the Westerschelde estuary, SW the Netherlands. Permission for the field work was issued by the Provincie Zeeland, the Netherlands (Directie Ruimte, Milieu en Water). The study site has a semi-diurnal tidal regime with a mean tidal range of 3.9 m and a yearly average salinity of 24. Experimental patches were randomly installed at a distance of at least 5 m from each other within a 50×50 m area at the middle, homogeneous, part of the flat (tidal elevation = +17.9 cm MSL, median particle size = 74.5 µm, mud content = 42%, sediment water content = 62%, sediment total organic matter = 3.9%). Five patches of sediment (16 m^2^) were covered with a polyethylene (1 mm) and tarpaulin (140 g. m-2) sheet [44], [45] for 40 days which mimicked the effects of algal mats covering the substratum, i.e. reduced exchange of oxygen with the water column and reduced light penetration. Our manipulation significantly depleted pore water oxygen and ammonium conditions, without affecting other sediment properties like water and mud content and the bulk availability of organic matter (One-way analysis of variance between undisturbed and disturbed sediments at opening of the plots on March 30th, 2005: p>0.05; Table S1). Oxygen penetration was very shallow, ranging between 0 and 1000 µm, with dissolved oxygen pore water concentration <2 mg.L-1, i.e. hypoxia (Figure S1). In addition, five patches were left undisturbed and used as controls to compare the recovery status of the disturbed sediments over the course of the experiment.

Three hypoxic patches were used to track the post-hypoxia variation in ecosystem properties and reassembling of the macrofauna community into detail, i.e. weekly during the first two weeks and subsequently biweekly until 25 weeks after hypoxia at the end of September 2005. In addition, the macrofauna community composition was determined in September 2006 (i.e. 78 weeks) as well. The analysed ecosystem properties were sediment bed level height (laser altimetry, n = 6.patch^−1^), sediment oxygen pore water concentration and penetration (Unisense OX 25 microelectrode, n = 2.patch^−1^), ammonium pore water concentration (SANplus segmented flow analyser, SKALAR, n = 2.patch^−1^), total organic matter (loss on ignition at 500°C for 2 hours, n = 2.patch^−1^) from the upper 10 cm of sediment, and chlorophyll a (Chl a) concentration (n = 2.patch^−1^). Chlorophyll a samples were collected with 3.6 cm inner diameter (i.d.) corers and stored in the dark on dry ice and subsequent at –80°C in the laboratory awaiting further analyses. Chlorophyll a concentrations were determined by HPLC analysis of the supernatant, extracted from the lyophilised sediment by adding 10 ml 90% acetone. The two other hypoxic patches were sampled one, 10 and 22 weeks after re-oxygenation to determine the recovery of rates of organic matter mineralization, sediment oxygen consumption, and denitrification. These three times reflect the major shifts in macrofauna diversity, community composition and dominant functional traits during post-hypoxia succession (see results). Furthermore, samples collected during these three occasions indicated that the temporal variation in macrofaunal communities did not differ among the five hypoxic patches.

In order to avoid disturbance in the plots due to repetitive sampling, samples were collected from a bridge, and sampling holes were filled with closed PVC tubes, pushed flush with the sediment surface. Furthermore, to minimise possible edge effects, sampling only occurred in the inner 3×3 m.

### Benthic Macrofauna Community

Macrofauna samples were randomly collected from each replicate patch during low tide with a corer (i.d. 12.5 cm) to a depth of 40 cm, fixed with a neutralized 8% formalin solution and subsequently washed over a 500 µm sieve. All individuals were sorted, counted, identified and grouped according to their feeding, motility and sediment reworking traits (Table S2). Bivalve biomass were obtained by determination of the ash free dry weight (4 h combustion at 450°C of dried individuals) and the biomass of other species was calculated by multiplying the organisms’ blotted wet weight with a species-specific ISO certified wet weight-ash free dry weight conversion factor [46].

### Measures of Ecosystem Processes

#### Nutrient flux rates

In order to determine nutrient flux rates, triplicate plexiglas cores of sediment (i.d. 10 cm) were randomly withdrawn without disturbing the sediment surface. The cores were pre-incubated for 48 hours in the dark at field temperature in a climate room within one hour after sampling. Preceding incubation, water was carefully added to the cores without creating suspension of the sediment (water constituted two thirds of the core) and cores were placed uncapped and submerged in an open tank containing aerated water from the Westerschelde estuary. Teflon coated magnets, rotated by a central magnet, were placed approximately 5 cm above the sediment surface to stir the water in order to avoid oxygen depletion at the sediment-water interface. All equipment used for the incubations was pre-incubated in Westerschelde water to avoid introduction of new surfaces for O_2_, N_2_ and Argon adsorption and desorption [47]. Sediment oxygen consumption and organic matter mineralization (i.e. dissolved inorganic carbon (DIC) release rates) were calculated as the difference in concentration between the start and end of incubation which lasted for 6–10 h, depending on the decrease of oxygen in the overlying water. Dissolved oxygen was determined using Winkler titration [48], samples for DIC were analyzed within 24 h by flow injection [49]. Samples for N_2_ and Argon concentrations were collected at 1.5–2 h time intervals during incubation and preserved in 20 µl HgCl_2_. Denitrification rates (i.e. N_2_ gas production rates) were determined within one week using membrane inlet mass spectrometry, normalized by those of Argon and calculated by linear regression, corrected for the refill water [50].

#### Bio-irrigation, primary production and bed load sediment transport

The bio-irrigation of the sediments was indirectly inferred from vertical sediment ammonium pore water profiles [51]. Therefore, two 6.2 cm i.d. corers, containing about 15 cm of sediment, were extracted from each patch without disturbing the sediment surface. Corers were sliced upon arrival in the laboratory and frozen at −20°C awaiting analysis. Recovery of deep irrigation of the sediment (5–10 cm) was assessed 0, 1, 6, 14 and 25 weeks after hypoxia. Bedload sediment transport rates (erosion, accretion) were calculated as the difference in bed level height between sampling occasions. The microalgal biomass, measured as Chl a concentration in the first 0.3 cm, was used as a proxy for benthic primary production [52].

### Data Analysis and Statistics

Temporal changes in ecosystem properties and processes in recovering and undisturbed sediments were assessed with Repeated Measures Analysis of Variance of transformed data, in which both Treatment (i.e. recovering vs. undisturbed sediments) and Time (i.e. weeks after hypoxia) were used as fixed factors. Proportional data were arcsine-squareroot transformed whereas a logarithmic (log_e_) transformation was applied to all other data. The homogeneity of the variance-covariance structure was analyzed using the Mauchley test of sphericity (Table S3), and Bartlett’s and Cochran’s tests were used to verify homogeneity of variances. If sphericity was not met, adjusted *F*-tests were applied based on the Greenhouse-Geisser corrections in order to interpret the significance of the within subject (i.e. repeated measure) effect. Replicated samples of variables per plot were pooled to avoid pseudoreplication. In order to evaluate recovery status of ecosystem processes and properties, planned contrasts between recovering and undisturbed sediments were performed at one, 10 and 22 weeks post-hypoxia, which encompass the time prior (April), during (June) and after (September) the natural recruitment period at our study site. These sampling occasions were thus deliberately chosen a priori since we anticipated that the structure and functional traits of the recolonizing community would differ among these three occasions. In this respect, we applied paired t-tests with separate error terms based on the two levels being compared, as is recommended for planned comparisons of repeated measures of properties over time in the same plots [53]. Since subsurface pore water ammonium concentrations (5–10 cm) were not available at 10 and 22 weeks post-hypoxia, recovery status for this property corresponding to the timing during and after the natural recruitment period was assessed respectively 14 and 25 weeks after hypoxia.

In order to understand how recovering macrofaunal assemblages contribute to post-disturbance ecosystem functioning, the role of species richness, community total biomass and abundance, and community-wide impact on sediment mixing (i.e. bioturbation) in explaining variation in ecosystem processes (i.e. denitrification, oxygen consumption, organic matter mineralization, bed load sediment transport, primary production, and bio-irrigation) among recovery stages was inferred using Distance based Linear Models (DistLM, [54]). The community potential to bioturbate (BPc) was calculated according to Solan et al. [18] taking into account the population biomass of each species based on the macrofauna samples and the species’ impact on sediment bioturbation through its specific mobility and sediment reworking traits (Table S2). The most reliable predictor for the variation in each ecosystem process in recovering and undisturbed sediments was inferred by applying the Akaike’s information criterion (AIC, [55]).

## Results

### Macrofauna Community Recovery

**Figure 1:**
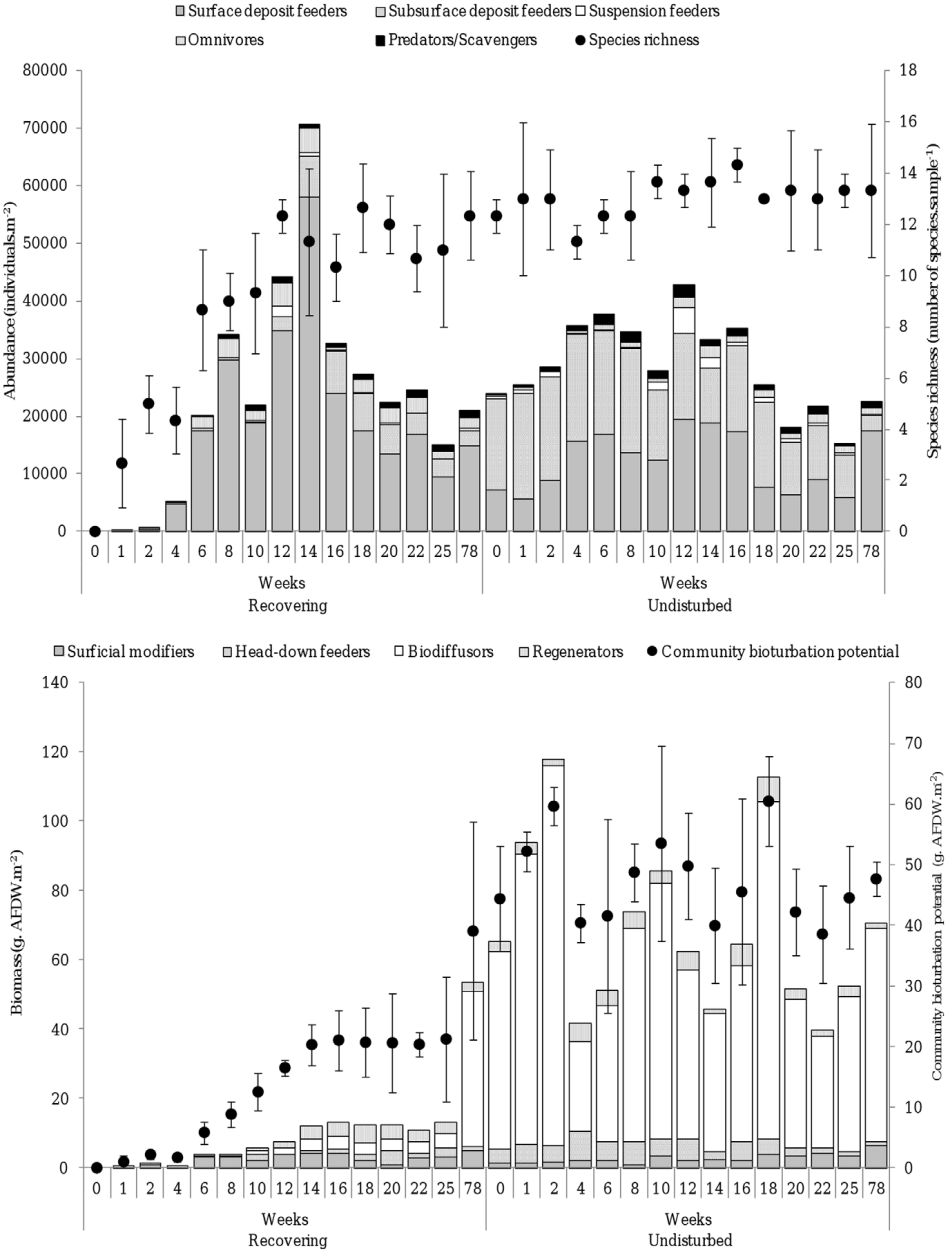
Temporal variation in macrofauna community properties in recovering and undisturbed sediments. Top panel; trophic group composition, abundance and species richness. Bottom panel; sediment reworking trait group composition, biomass and community bioturbation potential. Error bars represent ±95% confidence intervals.

Repeated measures analysis of variance revealed that macrofaunal diversity, total abundance, biomass and functional group composition significantly differed over time between recovering and undisturbed sediments (Table 1). Specifically, surface deposit feeding species that only modify the surface sediment layer abundantly colonized the disturbed sediments and dominated the community during the first 14 weeks following hypoxia. Afterwards, dominance shifted to head-down feeders and regenerators that actively transport sediment from depth to the surface, and to biodiffusors that randomly transport sediment particles over short distances (Figure 1). In contrast, deposit feeding and biodiffusing species always dominated the undisturbed community, indicating that the changes observed after hypoxia reflected successional dynamics imputable to recolonisation after disturbance (Figure 1). All species had successfully colonized the sediment and both species number and total abundance were restored after 22 weeks (species richness: *t* = 3.18, d.f. = 2, p = 0.086; total abundance: *t* = 0.34, d.f. = 2, p = 0.767). However, the recovering communities had significantly lower biomass (*t* = 6.37, d.f. = 2, *p* = 0.024) and less effects on sediment mixing (*t* = 6.36, d.f. = 2, p = 0.024) after 22 weeks of recovery as compared to the undisturbed communities (Figure 1).

**Table 1:** Results of Repeated Measures Analysis of Variance on ecosystem properties and processes during the experiment.

Ecosystem property or process	Treatment				Time				Treatment × Time			
	d.f.	MS	*F*	P	d.f.	MS	*F*	p	d.f.	MS	*F*	p
Species richness	1	1.029	556.60	<0.001	14	0.150	48.40	<0.001	14	0.130	41.97	<0.001
Total abundance	1	7.678	156.21	<0.001	14	2.382	123.31	<0.001	14	2.192	113.47	<0.001
Total biomass	1	23.820	1502.66	<0.001	14	0.323	7.21	<0.001	14	0.412	9.22	<0.001
Community bioturbation potential	1	10.442	4867.18	<0.001	14	0.339	30.71	<0.001	14	0.358	32.46	<0.001
Proportional biomass surficial modifiers	1	2.411	250.78	0.001	14	0.257	23.13	<0.001	14	0.341	30.72	<0.001
Proportional biomass head-down feeders	1	0.652	161.61	<0.001	14	0.016	1.33	0.221	14	0.028	2.26	0.016
Proportional biomass biodiffusors	1	20.059	1047.82	<0.001	14	0.094	5.28	<0.001	14	0.073	4.09	<0.001
Proportional biomass regenerators	1	0.020	0.83	0.415	14	0.059	4.51	<0.001	14	0.043	3.32	0.001
Proportional abundance surface deposit feeders	1	1.219	308.18	<0.001	14	0.258	20.81	<0.001	14	0.129	10.41	<0.001
Proportional abundance subsurface deposit feeders	1	3.584	616.97	<0.001	14	0.138	7.41	<0.001	14	0.099	5.32	<0.001
Proportional abundance suspension feeders	1	0.254	179.07	<0.001	14	0.020	11.02	<0.001	14	0.004	1.95	0.040
Proportional abundance omnivores	1	0.066	19.39	0.012	14	0.037	5.76	<0.001	14	0.011	1.75	0.070
Proportional abundance predators and scavengers	1	0.001	0.08	0.789	14	0.016	2.08	0.027	14	0.010	1.34	0.214
Chlorophyll a content	1	0.485	19.60	0.011	13	0.172	17.77	<0.001	13	0.073	7.57	<0.001
% Total organic matter	1	0.002	0.26	0.637	13	0.001	1.25	0.275	13	0.001	1.66	0.099
Pore water ammonium 0–1 cm	1	2.867	7.93	0.048	13	0.320	1.80	0.069	13	0.338	1.90	0.052
Pore water ammonium 5–10 cm	1	3.015	41.73	0.003	4	0.158	14.06	<0.001	4	0.040	3.55	0.030
Bed level height	1	0.039	22.37	0.009	11	0.002	11.27	<0.001	11	0.002	13.56	<0.001
Oxygen penetration depth	1	0.014	0.15	0.722	12	0.084	1.05	0.420	12	0.112	1.41	0.192
Denitrification	1	0.284	50.37	0.019	2	0.019	10.51	0.026	2	0.020	10.99	0.024
Total oxygen consumption	1	0.153	3.10	0.220	2	0.057	3.88	0.116	2	0.003	0.17	0.847
Re-oxidation of reduced compounds	1	0.001	0.08	0.801	2	0.016	1.47	0.333	2	0.002	0.20	0.830
Organic matter mineralisation*	1	0.051	1.05	0.413	2	0.098	4.35	0.172	2	0.018	0.80	0.466

* denotes adapted significance levels deduced from Greenhouse-Geisser corrections when sphericity assumption for repeated measures was not met. Data of bed level height were occasionally lacking for week 0 and week 12 and data of oxygen penetration depth for week 4.

### Recovery of Ecosystem Processes

**Figure 2:**
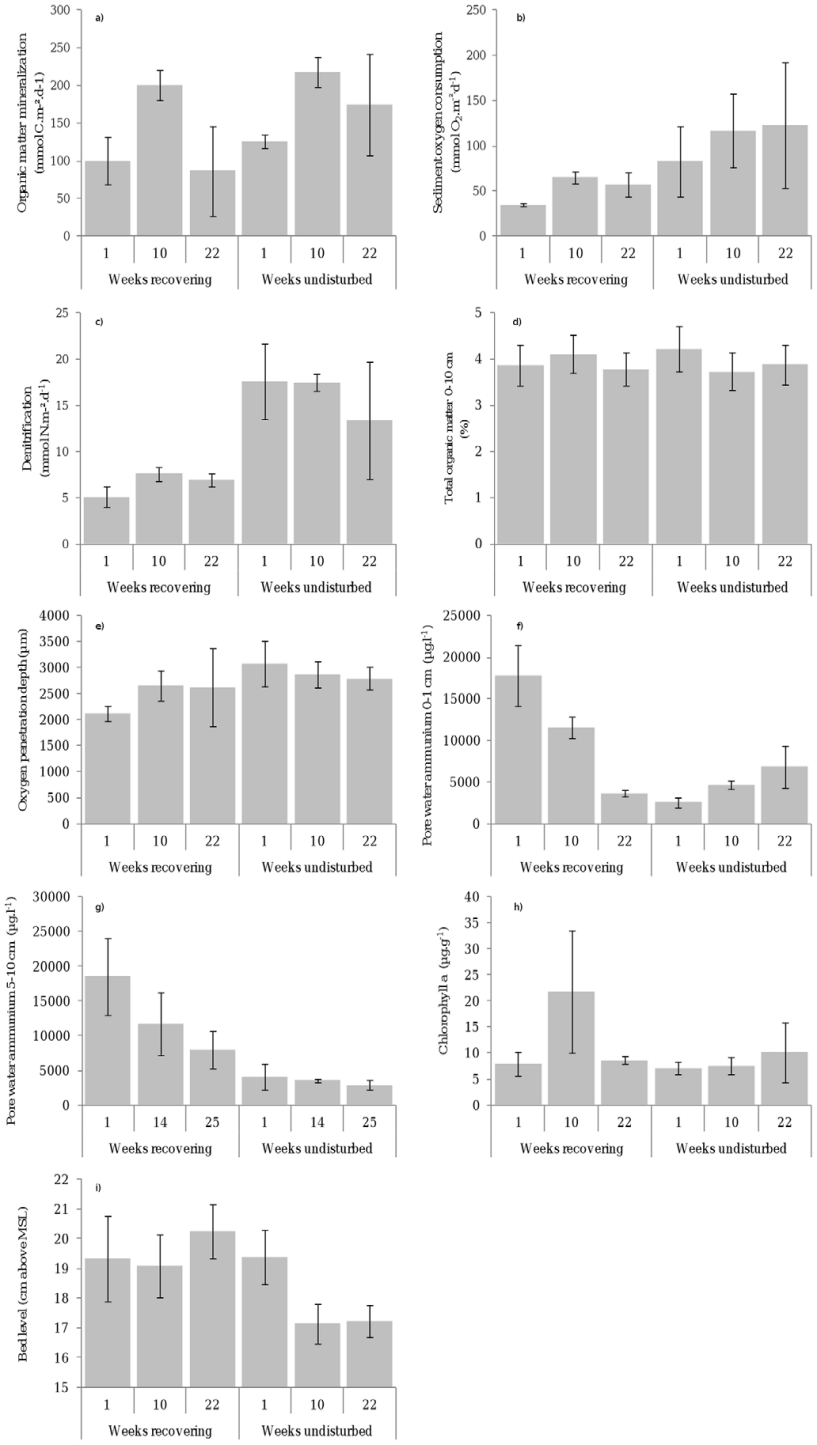
Variation in ecosystem processes and properties among recovery stages and in undisturbed sediments. (a) organic matter mineralization (DIC), (b) total sediment oxygen consumption, (c) denitrification, (d) percentage of total organic matter, (e) oxygen penetration, (f) pore water ammonium concentration in surface sediment, (g) ammonium pore concentration in deep sediments, (h) chlorophyll a concentration, and (i) sediment bed level height. Error bars represent ±95% confidence intervals.

Repeated measures analysis of variance indicated that denitrification, Chl a concentration, deep porewater ammonium concentration, and bed level height significantly differed over time between recovering and undisturbed sediments (Table 1). Oxygen consumption rates and removal rates of bioavailable nitrogen from the sediment as a consequence of denitrification were, respectively, only 42–56% and 29–52% of the rates recorded in the undisturbed sediments throughout the experiment (Figure 2b,c). Deep ammonium pore water concentrations in the sediment were enhanced in the disturbed sediments whereas dissolved inorganic carbon release rates from the sediment did not differ among recovering and undisturbed sediments at all times (Table 1, Figure 2a,g). Planned contrasts indicate that deep pore water ammonium concentrations and denitrification rates were still significantly reduced in recovering sediments after the natural recruitment period in September (pore water ammonium 5–10 cm: *t* = 19.98, d.f. = 2, p = 0.003; denitrification: *t* = 131.05, d.f. = 2, p = 0.005). Chlorophyll a concentration was significantly higher after 10 weeks in the recovering sediments (*t* = 4.57, d.f. = 2, *p* = 0.045) but differed no longer from the undisturbed sediment after 22 weeks in September (*t* = 2.79, d.f. = 2, *p* = 0.108) (Figure 2h). Using surface sediment Chl a concentrations and the regression equation provided for the Westerschelde estuary [56], average primary production is estimated to be 1.42 g C.m^−2^.day^−1^ during the first 12 weeks of recovery which is 78% higher than in the undisturbed sediments during the same period. Further, a net sediment bed erosion of 0.3±0.05 SD mm.day^−1^ took place in the undisturbed sediments from week 4 onwards until week 25, while the bed level remained more or less stable in the recovering sediments during that period (Figure 2i).

### Biodiversity – Ecosystem Process Relationships

Distance based linear models demonstrated that the variation in ecosystem processes in the recovering and undisturbed sediments was significantly explained by the macrofauna community for four out of the six processes that were measured and assessed: denitrification, primary production, deep bio-irrigation and bed load sediment transport (Table 2). Akaike’s information criterion identified BPc to be the best predictor of the variability in denitrification, bed level height, Chl a concentration, and deep porewater ammonium concentration. In contrast, higher AIC values show that species richness and community total abundance were in general of minor importance in explaining process variability among recovery stages as compared to community total biomass and BPc (Table 2, Figure S2). Species richness only significantly explained the variation in bed level height and deep porewater ammonium concentration in the recovering and undisturbed sediments, though clearly to a lesser extent than BPc. Especially a high proportion of the variation in subsurface processes, i.e. deep irrigation (78%) and denitrification (96%) was explained by BPc.

**Table 2:** Influence of macrofauna species richness, abundance, biomass, and bioturbation on the variability in ecosystem processes and properties in recovering and undisturbed sediments.

Ecosystem process	Macrofauna property	SS	F	p	R^2^	AIC
Denitrification^*^	Community bioturbation potential	147.06	105.41	0.0068	0.96344	3.57
	Total biomass	146.05	88.619	0.004	0.95681	4.56
	Total abundance	69.353	33.306	0.0416	0.45434	19.78
	Species richness	34.69	11.764	0.3734	0.22726	21.87
Sediment oxygen consumption^†^	Community bioturbation potential	4277	99.403	0.0526	0.71306	37.954
	Total abundance	2872.7	36.766	0.0754	0.47894	41.533
	Total biomass	2734.7	33.519	0.1554	0.45593	41.793
	Species richness	66.055	4.45E+02	0.8908	1.10E+02	45.378
Organic matter mineralisation^‡^	Total abundance	2990.9	1.019	0.4394	0.20303	49.474
	Community bioturbation potential	2851.2	0.95998	0.3424	0.19354	49.545
	Total biomass	1759.1	0.54242	0.4804	0.11941	50.073
	Species richness	1069.8	0.31324	0.7154	7.26E+02	50.384
Primary production^**^	Total biomass	12485	55.427	0.0254	0.17572	218.08
	Community bioturbation potential	12259	54.213	0.0262	0.17254	218.19
	Species richness	1230.4	0.4582	0.5252	1.73E+02	223
	Total abundance	843.23	0.31228	0.5804	1.19E+02	223.16
Deep bio-irrigation^††^	Community bioturbation potential	7.98E+12	28.256	0.0004	0.77934	173.33
	Species richness	5.96E+12	11.128	0.025	0.58178	179.73
	Total biomass	5.91E+12	10.939	0.0122	0.57759	179.83
	Total abundance	9.88E+11	0.85438	0.3822	9.65E+02	187.43
Bed load sediment transport^‡‡^	Community bioturbation potential	16.908	96.715	0.0062	0.30537	15.318
	Species richness	14.012	74.539	0.0094	0.25307	17.06
	Total biomass	12.423	63.639	0.0198	0.22437	17.965
	Total abundance	4.59E-03	1.82E+01	0.9658	8.29E-01	24.061

All distance based models were performed with 4999 random permutations. SS, explained sum of squares of the model; F, pseudo-*F* statistic; p, significance level; R^2^, the proportion of variance in the model which is explained by the predictor; AIC, Akaike’s information criterion. The best model for each process according to AIC is shown on top. Total model sum of squares: ^*^152.64, ^†^5998, ^‡^14732, ^**^71049, ^††^1.024 10^9^, ^‡‡^55.369; unexplained sum of squares of the model = SS_total_ – SS_model_. For details on the process measurements and assessments: see Methods.

## Discussion

### Engineering Macrofauna

Although our deliberately created hypoxic conditions were caused artificially, the patterns of species recolonisation and community assembly followed those observed for similar environments where hypoxia occurred naturally (for more details see [57]–[61]). Following re-oxygenation, the macrofaunal community increased in species richness and functional diversity along with increasing organism-sediment interactions by bioturbating large animals, as observed for large-scale organically-enriched sediments [62], [63]. Moreover, hypoxia did not change sediment metabolism; i.e. sediment organic matter and mineralization rates did not differ significantly among recovering and undisturbed sediments throughout the experiment (Table 1; Fig. 2a,d). Hence, we are convinced that our findings are not biased by our method of inducing hypoxia, nor by the effects of sediment metabolism on macrofauna and, consequently, enables us to properly investigate the relation between macrofaunal reassembling diversity and functional traits (i.e. bioturbation) and the recovery of ecosystem processes.

Four out of the six ecosystem processes that were measured and assessed were related to the variability in macrofaunal community characteristics: denitrification, bio-irrigation, primary production and bed load sediment transport. Both denitrification rates and deep pore water ammonium concentrations were not recovered after six months of recovery when all species had recolonised the disturbed sediments. The variability in denitrification and deep pore water ammonium concentrations, as a proxy for deep bio-irrigation, was mainly explained by the changes in bioturbation potential, while species richness and total abundance were in general of minor importance in statistically explaining these process variabilities. Several studies have shown that bioturbation activity increases denitrification, e.g. [64], [65]. At an early stage of recovery, the lack of irrigation and particle mixing to deep sediment layers due to the absence of large bioturbating animals very likely impaired the development of aerobic zones and redox interfaces needed for coupled nitrification-denitrification which may explain the slow recovery of fixed nitrogen removal from the sediment. Furthermore, the rapid recovery of oxygen penetration depth and surface pore-water ammonium concentrations (Figure 2e,f) due to molecular oxygen diffusion into the upper surface layer, in comparison with the slow recovery of pore water ammonium concentrations at depth, suggest that the recovery of benthic oxygen consumption was mainly hampered by the limited re-oxydation of reduced compounds that have accumulated at depth. Moreover, the low respiratory coefficient (ratio between O_2_ consumption and dissolved inorganic carbon (DIC) release) in early recovering sediments (<0.4, and respectively 45 and 38% lower in comparison with the undisturbed sediments after one and 10 weeks of recovery) further illustrates a limited re-oxydation of reduced compounds at early stages of recovery [66], [67]. In addition, the variability in primary production and bed load transport was also best explained by the community bioturbation potential, though to a lesser extent as for subsurface processes, i.e. denitrification and bio-irrigation. The observed resistance to sediment erosion and enhanced primary production in early recovering sediments likely relates to the absence of intense bioturbation. Intense bioturbation, as did occur in the undisturbed sediments, increases bottom roughness and enhances erosion of the sediment and attached benthic microalgae [68], [69]. This inhibits the positive feedback interactions between growth of benthic microalgae (i.e. primary production) and sediment stability [70].

### Successional States and Ecosystem Resilience

Multiple human pressures have resulted in a significant and rapid decline in biodiversity on a global scale [71], [72], which has stimulated the research of the relationship between diversity and ecosystem functioning over the past 15 years. Species diversity seems particularly instrumental to ecosystem functioning in more diverse systems [73], [74] where environmental factors become less important in modifying ecosystem processes [73], [75]. In this study, macrofauna species richness was relatively low and organism-sediment interactions (i.e. bioturbation) were particularly important in mediating return rates of several ecosystem processes. For example, the return rates of mainly microbial-mediated processes such as denitrification and oxygen consumption relate to the slow return rate of macrofaunal bioturbation of the sediment, despite the limited effect of hypoxia on bacterial activities (assessed as DIC rates, Figure 2a). Large organisms that create environmental heterogeneity in deep layers through bioturbation –and bio-irrigation activities [32] are typically present during late recovery stages. This study indicates that these organisms influence ecosystem processes disproportionately more than their juvenile and highly abundant, smaller life-stages during early recovery stages or community-wide species richness. Similarly, Bolam et al. [14] found that particularly the abundance of the active and largest species at their study site, *Nepthys hombergii*, drove the relation between the benthos community and oxygen consumption in a field experiment where benthos diversity and biomass was manipulated.

Hypoxia occurs on different time and spatial scales, ranging from large areas with persistent oxygen deficiency (e.g. oceanic oxygen minimum zones) to small-scale, localized single events, but recurring episodic, periodic or seasonal events are a common feature [76]. Due to the slow growth of species with strong effects on sediment particle reworking, macrofaunal bioturbation only restored in September 2006; i.e. after two growing seasons (Figure 1). Consequently, the slow re-instalment of such organism-sediment interactions is very likely to impair the resilience of systems that are prone to seasonal or recurring hypoxic events because such iterative events do not allow complete recovery of the functionally important large, strong bioturbating and bio-irrigating species before hypoxia reoccurs. The different return rates of hypoxic events and ecosystem engineering activities may therefore lock the system in a state where only small opportunistic species persist that have rather limited effects on biogeochemical cycling (reviewed in [25]), in particular re-oxidation of anoxic sediments and consumption of organic matter. Such conditions likely invoke oxygen depletion through enhanced respiratory demands and such legacy effects have been suggested to contribute to increased hypoxia in Chesapeake Bay, the Gulf of Mexico and the Baltic Sea [77]–[79]. Our results indicate that this degraded state will only be reversed if the re-supply of oxygen rich water lasts long enough to re-establish large-sized macrofaunal organisms with strong engineering effects on the reduced sediment.

## Supporting Information

Figure S1 **Top panel: Oxygen penetration in the undisturbed sediments and the disturbed sediments at opening of the plots on March 30^th^, 2005.** Bottom panel: Temporal variation of pore water oxygen concentration at 500 µm depth in the recovering and undisturbed sediments. Error bars represent one standard error.
(DOCX)

Figure S2 **Scatterplots showing relationships between species richness, total abundance, total biomass and community bioturbation potential and ecosystem processes.** Filled symbols indicate significant relations at p<0.05, as deduced from Distance based Linear Models.
(DOCX)

Table S1 **Effects of the applied defaunation method on abiotic properties.** Significance levels of comparisons between undisturbed and disturbed sediments (n = 3) at day 0 after removing of the sheets are obtained from One-way Analysis of Variance (ANOVA).
(DOCX)

Table S2 **Macrobenthos species present in the recovering and undisturbed sediments at the study site (Paulinapolder, 51° 21′ 24′′ N, 3° 42′ 51′′ E).** Feeding traits (surface deposit feeder (SDF), subsurface deposit feeder (SSDF), suspension feeder (SF), omnivores (O), predators/scavengers (P), motility traits (living in a fixed tube (T), limited movement (L), slow movement through the sediment (S) and free movement via burrow system (F)) and sediment reworking traits (surficial modifiers (SM), head-down feeders that actively transport sediment to the surface (HD), biodiffusors that randomly transport sediment over short distances (B), and regenerators that excavate holes and transfer sediment from depth to the surface (R)) are indicated. Species traits were retrieved from Fauchauld and Jumars (1979) Oceanography and Marine Biology an Annual Review 17∶193–284, Gerino et al. (2003) Vie et Milieu 53∶221–232, Volkenborn and Reise (2007) Journal of Sea Research 57∶78–88, www.marlin.ac.uk, and own observations from stable isotope and luminophore tracer studies; e.g. Rossi et al. (2009) Oikos 118∶503–512, Montserrat et al. (2009) Estuarine Coastal and Shelf Science 83∶379–391
(DOCX)

Table S3 **Results of Mauchley tests for sphericity.**
(DOCX)
