# Supplementary material for: Organism-Sediment Interactions Govern Post-Hypoxia Recovery of Ecosystem Functioning
Source: PLoS One. 2012 Nov 21;7(11):e49795. doi: 10.1371/journal.pone.0049795 (PMC3504103; doi:10.1371/journal.pone.0049795)
Supplement: Figure S2 — Scatterplots showing relationships between species richness, total abundance, total biomass and community bioturbation potential and ecosystem processes. Filled symbols indicate significant relations at p<0.05, as deduced from Distance based Linear Models. (DOCX) [file pone.0049795.s002.docx]

**Figure S2**

**Figure S2 *continued***
