# Supplementary material for: Organism-Sediment Interactions Govern Post-Hypoxia Recovery of Ecosystem Functioning
Source: PLoS One. 2012 Nov 21;7(11):e49795. doi: 10.1371/journal.pone.0049795 (PMC3504103; doi:10.1371/journal.pone.0049795)
Supplement: Table S1 — Effects of the applied defaunation method on abiotic properties. Significance levels of comparisons between undisturbed and disturbed sediments (n = 3) at day 0 after removing of the sheets are obtained from One-way Analysis of Variance (ANOVA). (DOCX) [file pone.0049795.s003.docx]

**Table S1**

| Sediment property | *F* | *p* | *p(levene)* |
| --- | --- | --- | --- |
|  |  |  |  |
| Total organic matter | 1.8140 | 0.2493 | 0.1856 |
| Oxygen penetration depth | 17.3571 | 0.0141 | 0.1012 |
| Ammonium 0-1 cm | 5.3166 | 0.0824 | 0.0717 |
| Ammonium 1-2 cm | 31.0034 | 0.0051 | 0.1264 |
| Ammonium 2-3cm | 21.6594 | 0.0096 | 0.3082 |
| Ammonium 3-5cm | 201.1591 | 0.0001 | 0.1706 |
| Ammonium 5-10 cm | 5.2563 | 0.0836 | 0.0834 |
| Mud content (<63µm) 0-1 cm | 0.8969 | 0.3972 | 0.1783 |
| Water content (%) 0-1 cm | 0.1262 | 0.7403 | 0.3607 |
|  |  |  |  |
