# Supplementary material for: Organism-Sediment Interactions Govern Post-Hypoxia Recovery of Ecosystem Functioning
Source: PLoS One. 2012 Nov 21;7(11):e49795. doi: 10.1371/journal.pone.0049795 (PMC3504103; doi:10.1371/journal.pone.0049795)
Supplement: Table S2 — Macrobenthos species present in the recovering and undisturbed sediments at the study site (Paulinapolder, 51° 21′ 24′′ N, 3° 42′ 51′′ E). Feeding traits (surface deposit feeder (SDF), subsurface deposit feeder (SSDF), suspension feeder (SF), omnivores (O), predators/scavengers (P), motility traits (living in a fixed tube (T), limited movement (L), slow movement through the sediment (S) and free movement via burrow system (F)) and sediment reworking traits (surficial modifiers (SM), head-down feeders that actively transport sediment to the surface (HD), biodiffusors that randomly transport sediment over short distances (B), and regenerators that excavate holes and transfer sediment from depth to the surface (R)) are indicated. Species traits were retrieved from Fauchauld and Jumars (1979) Oceanography and Marine Biology an Annual Review 17∶193–284, Gerino et al. (2003) Vie et Milieu 53∶221–232, Volkenborn and Reise (2007) Journal of Sea Research 57∶78–88, www.marlin.ac.uk, and own observations from stable isotope and luminophore tracer studies; e.g. Rossi et al. (2009) Oikos 118∶503–512, Montserrat et al. (2009) Estuarine Coastal and Shelf Science 83∶379–391 (DOCX) [file pone.0049795.s004.docx]

**Table S2**

| Species | Feeding trait | Mobility | Sediment reworking |
| --- | --- | --- | --- |
|  |  |  |  |
| *Abra tenuis* | SDF | L | SM |
| *Aphelochaeta marioni* | SDF | L | SM |
| *Capitella capitata* | SSDF | L | HD |
| *Cerastoderma edule* | SF | S | B |
| *Eteone longa* | P | F | R |
| *Gammarus spp.* | SDF | S | SM |
| *Heteromastus filiformis* | SSDF | L | HD |
| *Hydrobia ulvae* | SDF | F | SM |
| *Macoma balthica* | SDF | L | B |
| *Malacoceros tetracerus* | SDF | L | SM |
| *Nemertinae spp.* | P | S | R |
| *Nereis diversicolor* | O | F | R |
| *Oligochaeta* | SSDF | S | HD |
| *Polydora ligni* | SDF | T | SM |
| *Pygospio elegans* | SDF | T | SM |
| *Retusa obtusa* | P | F | SM |
| *Scrobicularia plana* | SDF | L | B |
| *Streblospio benedicti* | SDF | L | SM |
|  |  |  |  |
