# Supplementary material for: Organism-Sediment Interactions Govern Post-Hypoxia Recovery of Ecosystem Functioning
Source: PLoS One. 2012 Nov 21;7(11):e49795. doi: 10.1371/journal.pone.0049795 (PMC3504103; doi:10.1371/journal.pone.0049795)
Supplement: Table S3 — Results of Mauchley tests for sphericity. (DOCX) [file pone.0049795.s005.docx]

**Table S3**

| Ecosystem property or process | W | χ² | df | p |
| --- | --- | --- | --- | --- |
|  |  |  |  |  |
|  |  |  |  |  |
| Species richness | 3.758266E+17 | 34.68678 | 104 | 1.000000 |
| Total abundance | 7.605204E+09 | 19.50180 | 104 | 1.000000 |
| Total biomass | 474.0187 | 5.281069 | 104 | 1.000000 |
| Community bioturbation potential | 1.074378E+11 | 21.77158 | 104 | 1.000000 |
| Proportional biomass surficial modifiers | 2.480009E+13 | 57.27776 | 104 | 0.999944 |
| Proportional biomass head-down feeders | 1.320021E+09 | 18.00078 | 104 | 1.000000 |
| Proportional biomass biodiffusors | 227749746 | 16.49465 | 104 | 1.000000 |
| Proportional biomass head-down regenerators | 1.480743E+09 | 18.09927 | 104 | 1.000000 |
| Proportional abundance surface deposit feeders | 1.758762E+09 | 18.24675 | 104 | 1.000000 |
| Proportional abundance subsurface deposit feeders | 6.767443E+09 | 19.40176 | 104 | 1.000000 |
| Proportional abundance suspension feeders | 2.996066E+19 | 38.43979 | 104 | 1.000000 |
| Proportional abundance omnivores | 6.828159E+13 | 27.30400 | 104 | 1.000000 |
| Proportional abundance predators and scavengers | 3.038895E+13 | 26.61009 | 104 | 1.000000 |
| Microalgal biomass | 1.708973E+12 | 14.80569 | 90 | 1.000000 |
| % Total organic matter | 1.478932E+22 | 26.83302 | 90 | 1.000000 |
| Pore water ammonium 0-1 cm | 4.882473 | 0.833484 | 90 | 1.000000 |
| Pore water ammonium 5-10 cm | 0.094802 | 5.693588 | 9 | 0.770150 |
| Bed level height | 4.832542E+20 | -6.49460 | 65 | 1.000000 |
| Oxygen penetration depth | 0.000020 | -2.10777 | 77 | 1.000000 |
| Denitrification | 0.333909 | 1.096886 | 2 | 0.577849 |
| Total oxygen consumption | 0.138708 | 1.975383 | 2 | 0.372436 |
| Re-oxidation of reduced compounds | 0.084997 | 2.465137 | 2 | 0.291543 |
| Organic matter mineralisation | 0.001888 | 6.272381 | 2 | 0.043448 |
|  |  |  |  |  |
